# First Reported Case of Neuroleptospirosis Complicated With Anton's Syndrome

**DOI:** 10.3389/fneur.2018.00966

**Published:** 2018-12-04

**Authors:** Nasheeda Saeed, Ching Soong Khoo, Rabani Remli, Zhe Kang Law, Petrick Periyasamy, Syazarina Sharis Osman, Hui Jan Tan

**Affiliations:** ^1^Neurology Unit, Department of Medicine, Universiti Kebangsaan Malaysia Medical Centre, Kuala Lumpur, Malaysia; ^2^Infectious Disease Unit, Department of Medicine, Universiti Kebangsaan Malaysia Medical Centre, Kuala Lumpur, Malaysia; ^3^Department of Radiology, Universiti Kebangsaan Malaysia Medical Centre, Kuala Lumpur, Malaysia

**Keywords:** neuroleptospirosis, seizures, cortical blindness, anton's syndrome, electroencephalogram

## Abstract

Leptospirosis is a spirochetal zoonotic disease with a wide clinical spectrum, often underdiagnosed especially when presented as an acute neurological manifestation. We report a case of a 24-year-old man with serologically positive leptospirosis, who presented with altered sensorium, seizures and subsequently developed cortical blindness. His brain MRI revealed bilateral occipital and later parietal lobe cerebritis.

## Introduction

Leptospirosis is considered the most common zoonotic disease in the world and is associated with major health impacts in developing countries (1). It has gained attention worldwide as an emerging infectious disease due to the 1995 Nicaragua epidemic of severe pulmonary hemorrhage syndrome, and identification of the disease among the US inner-city homeless population (1, 2). Leptospirosis is often under-recognized because of its varied and wide clinical manifestations, which can range from asymptomatic infection to fulminant or fatal outcome.

It is uncommon for leptospirosis to present as a primary neurological disease and the diagnosis can be missed because of its atypical presentation. Neurological manifestations can be diverse and are seen in about 10–15% of the cases, which are variable and remain unrecognized. Patients with atypical presentation are often empirically treated for cerebral malaria, tuberculous meningitis, hepatic encephalopathy, viral encephalitis depending on the seasonal prevalence, endemicity of the infective agent and clinical bias (3).

This paper describes a man from the People's Republic of China, who presented to us with seizures, and was later intubated for status epilepticus. He was extubated after a day and subsequently on day 7 of illness developed bilateral cortical blindness due to bilateral occipital cerebritis.

## Background

A 24-year-old man from China, pursuing higher education in Malaysia for the past 3 years with no known medical illnesses, presented to the Emergency Department (ED) with reduced consciousness. His roommate reported history of jerky movements of the whole body with drooling of saliva prior to becoming unconscious. There was no history of up-rolling of eye balls or sphincteric incontinence. On further history, he had flu-like illness with fever, myalgia and headache for one day. No behavioral changes were noticed preceding the event. There was no high-risk behavior, history of illicit drug use, smoking or alcohol abuse. He last travelled to China 3 months prior to this incident.

On examination, he had a Glasgow Coma Scale (GCS) of 7 (E1V1M5). No neck stiffness was elicited. His nervous system examination revealed a normal tone in all 4 limbs with 1+ reflexes; and bilateral flexor plantar response. Both his pupils were 2 mm equal and reactive to light. Cardiovascular, respiratory and abdomen examinations were unremarkable.

His vital signs revealed a pulse rate of 112 beats per minute, blood pressure of 128/50 mmHg, temperature of 37.1°C and oxygen saturation of 95% at room air. The blood glucose was 12 mmol/L. He was clinically well hydrated with no pedal edema, clubbing, cyanosis, jaundice, skin lesions or bruising. There was no eschar seen. He developed multiple episodes of generalized tonic clonic seizures without regaining consciousness despite three boluses of intravenous (IV) diazepam. He was then intubated and loaded with IV phenytoin at 15 mg/kg. He was normotensive throughout this event and his hospital stay.

His urine toxicology was negative and brain computed tomography (CT) did not reveal any abnormalities. Blood investigations showed leucocytosis, transaminitis, acute kidney injury with metabolic acidosis and myositis. Electrocardiogram (ECG) showed sinus tachycardia and his chest radiogram was normal. He was empirically treated with IV ceftriaxone and IV acyclovir for acute meningoencephalitis.

Apart from a mildly elevated protein of 0.75 g/l (normal value 0.2–0.4 g/L), his cerebrospinal fluid (CSF) analysis was essentially normal. The CSF was clear in appearance with normal pressure. No red blood cells and white blood cells were seen in the CSF. Cerebrospinal fluid glucose was 4.9 mmol/l. Indian Ink, Gram stain, AFB stain, qualitative globulins and cryptococcal antigen were all negative. Haemophilus influenzae type B, Neisseria menigitidis, group B Streptococcus and Streptococcus pneumoniae were not detected in the CSF.

**Figure 1:**
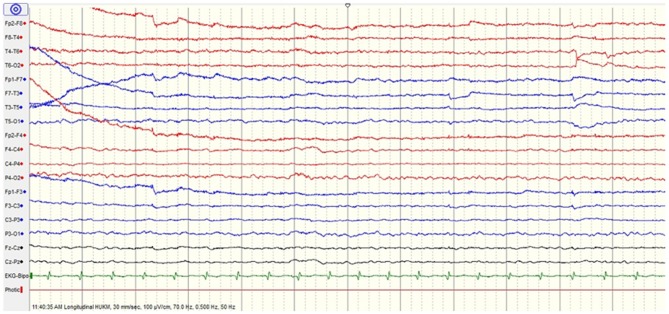
Initial electroencephalogram (EEG) shows mild encephalopathy.

He was subsequently extubated after 2 days. He remained hemodynamically stable with full GCS. Electroencephalogram (EEG) showed mild encephalopathy (Figure 1). On the 7th day of hospitalization, he complained of sudden blurring of vision. He became restless and aggressive, followed by a focal seizure (head turned to the left with facial twitching and jerky movements of the left upper limb). On examination, he was hemodynamically stable, and could move his four limbs. He was unable to count fingers, recognize color or perceive light even though he remained adamant that he could see (Anton's syndrome). Ophthalmologic examination revealed normal assessment of both eyes with no nystagmus or ophthalmoplegia.

**Figure 2:**
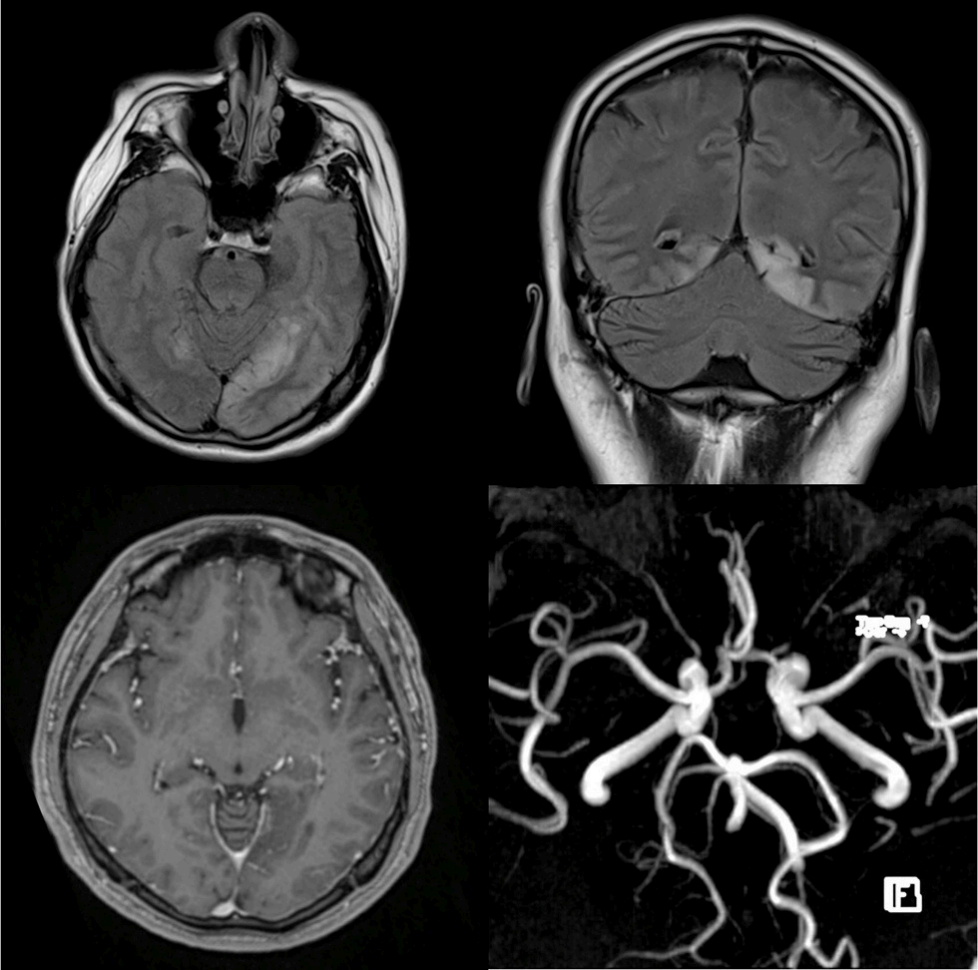
T2-weighted FLAIR MRI images on the axial (top left) and coronal (top right) planes exhibit abnormal hyperintense signals involving the cortices of both occipital lobes consistent with encephalitis. T1-weighted post gadolinium shows no enhancement of the affected occipital lobe (bottom left) and MR angiogram (MRA) is normal with no evidence of vasculitic changes (bottom right).

An urgent brain CT revealed a new hypodensity at the left occipital region. T2-weighted FLAIR MRI image showed abnormal hyperintense signals involving the cortices of both occipital lobes consistent with encephalitis. There was no restricted diffusion on DWI sequences. T1-weighted post gadolinium showed no enhancement of the affected occipital lobe and MR Angiography (MRA) was normal with no evidence of vasculitic changes (Figure 2). No MR venography was done. However, gadolinium study did not reveal any filling defect in the superior sagittal sinus to suggest thrombosis.

**Figure 3:**
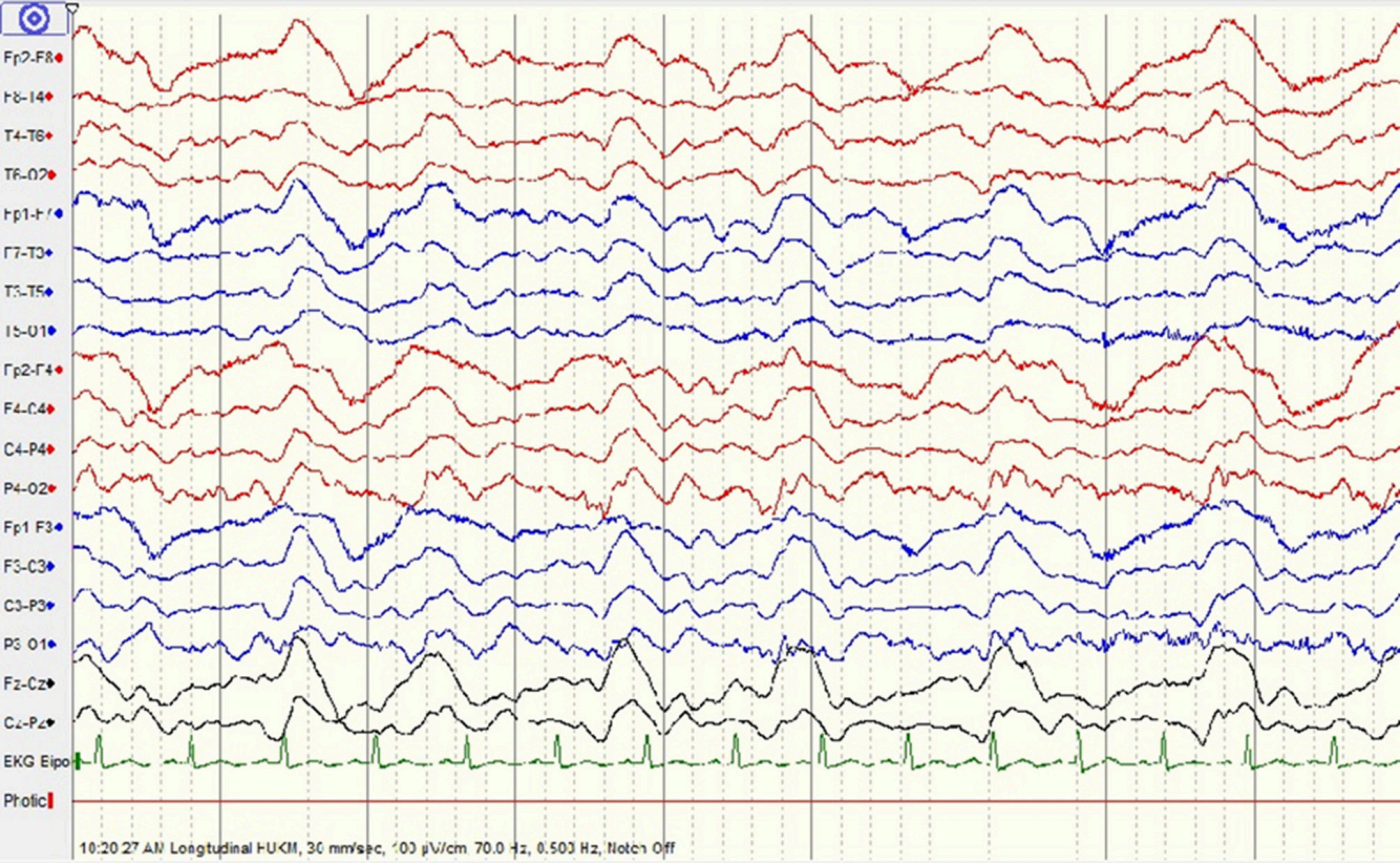
Repeated electroencephalogram (EEG) shows moderate encephalopathy with generalized periodic epileptiform discharges.

At this juncture, his serum leptospira IgM was detected. Microscopic agglutination test (MAT) for leptospirosis was pending. A diagnosis of neuroleptospirosis was made and he was treated with IV methylprednisolone 500 mg once daily for 5 days. Patient did not improve and displayed fluctuating consciousness level. His repeated EEG demonstrated moderate encephalopathy with generalized periodic epileptiform discharges (Figure 3). Pending confirmation of serum leptospira MAT, we started IV Immunoglobulin 0.4 g/kg for 5 days to treat for other possible causes such as limbic encephalitis.

**Figure 4:**
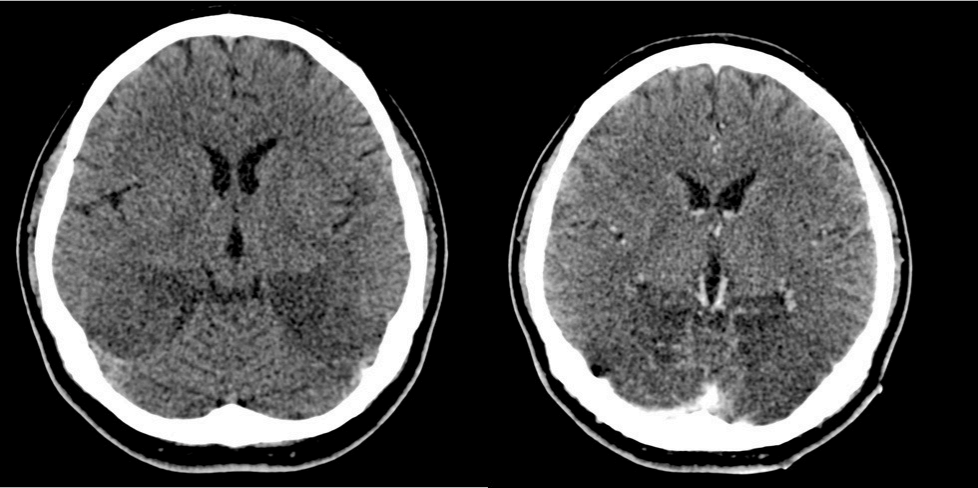
Plain (top left) and contrasted (top right) CT scans show progressive/enlarging hypodense encephalitic changes in both occipital lobes.

One week after the onset of blindness, plain and contrasted CT scans of the brain were done as there was no clinical improvement after treatment with methylprednisolone for 5 days. The repeated CT brain showed involvement of high parietal lobes bilaterally which were not seen in the previous imaging; and progressive/enlarging hypodense encephalitic changes in both occipital lobes (Figure 4).

Previous investigations including connective tissue disease screening and tumor markers were negative. His leptospiral MAT result was positive at 1:400. Both anti-N-methyl-D-aspartate receptor (NMDA) and Japanese encephalitis antibodies were negative. Rickettsia serology was negative. There was no eschar found. Scrub typhus is not prevalent in this part of Malaysia. It is mostly found in rural areas of Malaysia since early 1970s, with antibody prevalence to O. Unfortunately CSF Leptospira PCR or MAT was not available in our center, hence it was not done. We were unable to contact the patient for follow-up because he had gone back to his home country–China.

## Discussion

It is uncommon for leptospirosis to present primarily as a neurological disease. Neurological manifestations are seen in about 10–15% of patients with leptospirosis, and often remain unrecognized (3). From a study by Panicker et al. (4) on patients admitted to the general medical ward over a 3-year period with acute neurological disease, 40% of them were found to have leptospirosis. The most common manifestation was aseptic meningitis (4). Other neurological manifestations were myeloradiculopathy, myelopathy, Guillain-Barré syndrome like presentation, meningoencephalitis, intracerebral bleed, cerebellar dysfunction, iridocyclitis and tremor/rigidity.

Mathew et al. concluded from his study on 31 patients treated with leptospirosis that the common neurological presentations were altered sensorium, deeply comatose state and acute symptomatic seizures (3).

Our patient presented with status epilepticus; and on the 7th day of illness developed cortical blindness. Review of previous literature did report blindness due to uveitis; however, no reports pertaining to cortical blindness with subsequent deterioration and progressive neurological symptoms were documented.

The neuroimaging in our patient demonstrated bilateral occipital cerebritis. Development of new visual symptoms corresponded to the new MR findings, which were absent one week earlier when the seizures had occurred. The patient was normotensive throughout his stay which made Posterior Reversible Encephalopathy Syndrome (PRES) very unlikely. It is also unlikely that these were demyelination changes as demyelination changes are periventricular, juxta-cortical, and are not associated with cortical swelling.

Bilateral cortical blindness due to bilateral occipital cerebritis as a manifestation of neuroleptospirosis was not documented in any available literature. The CT findings in the cases by Mathew et al. (3) were diffuse cerebral edema, right middle cerebral artery territory infarct, and lacunar infarct in the left internal capsule. Sixty-seven percent of the cases had normal CT findings. A German group reported a case of neuroleptospirosis involving the basal ganglia (5).

Though primary neuroleptospirosis is uncommon, various neurological manifestations in leptospirosis have been described, including stroke, cerebral venous thrombosis, cerebral arteritis, subarachnoid hemorrhage, blindness due to uveitis, optic neuritis, transverse myelitis, cranial nerve palsy, Guillain-Barré syndrome, mononeuritis multiplex, peripheral nerve palsy, psychosis, suicidal behavior, cerebellitis, encephalitis, meningitis, chronic meningitis, and primary meningitis (6–10).

After an incubation period of 5 to 14 days on average, leptospirosis follows a biphasic nature with an initial leptospiremic phase lasting a week and an immune phase. It is likely that our patient developed the blindness during the immune phase of the illness. What exactly triggered the process causing the blindness despite treatment with appropriate antibiotic for 1 week is not known. A review by Alan et al. (1) suggests an underlying immunopathogenic process with disease determinants related to the inoculum size, host factors and virulence factors (1).

The prognosis of neuroleptospirosis is largely unknown. Most studies report mortality rates for systemic leptospirosis, varying from 5 to 15% (3). Altered sensorium, seizures and raised CSF protein were found to have a worse prognosis (3, 4) as in our case. The role of steroids in the treatment of leptospirosis during the immune phase is controversial. Based on the clinical experience, the use of steroids is found to be effective in reducing the severity and duration of illness (3, 4).

## Conclusion

Altered sensorium and seizures can be the primary manifestation of leptospirosis with minimal involvement of other organs, as demonstrated in our case.

Clinicians should have a high index of suspicion of this uncommon, diverse and delayed manifestation of leptospirosis among patients from an endemic area. Our patient developed bilateral cortical blindness due to leptospira infection with evidence of bilateral parieto-occipital cerebritis and progressive deterioration despite adequate antibiotic and steroid treatment.

## Author Contributions

NS, CK, RR, and ZL drafted the manuscript and reviewed the literature. PP and SO edited manuscript. HT reviewed manuscript.

### Conflict of Interest Statement

The authors declare that the research was conducted in the absence of any commercial or financial relationships that could be construed as a potential conflict of interest.
